# Evaluation of dosimetric misrepresentations from 3D conventional planning of liver SBRT using 4D deformable dose integration

**DOI:** 10.1120/jacmp.v15i6.4978

**Published:** 2014-11-08

**Authors:** Unjin A. Yeo, Michael L. Taylor, Jeremy R. Supple, Shankar Siva, Tomas Kron, Daniel Pham, Rick D. Franich

**Affiliations:** ^1^ School of Applied Sciences and Health Innovations Research Institute RMIT University Melbourne VIC Australia; ^2^ Radiation Oncology Victoria Genesis Care Melbourne VIC Australia; ^3^ Physical Sciences Peter MacCallum Cancer Center East Melbourne VIC Australia; ^4^ Sir Peter MacCallum Department of Oncology University of Melbourne Melbourne VIC Australia

**Keywords:** organ deformation, DIR, deformable dose accumulation, dose warping, 4D evaluation, liver SBRT

## Abstract

The purpose of this study is to evaluate dosimetric errors in 3D conventional planning of stereotactic body radiotherapy (SBRT) by using a 4D deformable image registration (DIR)‐based dose‐warping and integration technique. Respiratory‐correlated 4D CT image sets with 10 phases were acquired for four consecutive patients with five liver tumors. Average intensity projection (AIP) images were used to generate 3D conventional plans of SBRT. Quasi‐4D path‐integrated dose accumulation was performed over all 10 phases using dose‐warping techniques based on DIR. This result was compared to the conventional plan in order to evaluate the appropriateness of 3D (static) dose calculations. In addition, we consider whether organ dose metrics derived from contours defined on the average intensity projection (AIP), or on a reference phase, provide the better approximation of the 4D values. The impact of using fewer (<10) phases was also explored. The AIP‐based 3D planning approach overestimated doses to targets by 1.4% to 8.7% (mean 4.2%) and underestimated dose to normal liver by up to 8% (mean −5.5%; range −2.3% to −8.0%), compared to the 4D methodology. The homogeneity of the dose distribution was overestimated when using conventional 3D calculations by up to 24%. OAR doses estimated by 3D planning were, on average, within 10% of the 4D calculations; however, differences of up to 100% were observed. Four‐dimensional dose calculation using 3 phases gave a reasonable approximation of that calculated from the full 10 phases for all patients, which is potentially useful from a workload perspective. 4D evaluation showed that conventional 3D planning on an AIP can significantly overestimate target dose (ITV and ${\text{GTV}}_{+5\text{mm}}$), underestimate normal liver dose, and overestimate dose homogeneity. Implementing nonadaptive quasi‐4D dose calculation can highlight the potential limitation of 3D conventional SBRT planning and the resultant misrepresentations of dose in some regions affected by motion and deformation. Where the 4D approach is unavailable, contouring on the full expiration phase may yield more accurate dose calculations, most relevant in the case of the healthy liver, but the absolute dose differences are in general small for the other healthy organs. The technique has the potential to quantify under‐ and over‐dosage and improve treatment plan evaluation, retrospective plan analysis, and clinical outcome correlation.

PACS numbers: 87.55.‐x, 87.55.D‐, 87.55.de, 87.55.dk, 87.55.Qr, 87.57.nj

## I. INTRODUCTION

The presence of anatomic changes, involving organ deformation due mainly to respiratory motion, as well as filling and emptying of bladder, rectum, stomach, etc., may introduce discrepancies between planned and delivered doses in radiotherapy (RT). This could result in significant underdosing of target volumes and increased doses to healthy tissues, particularly for highly conformal techniques applied to thoracic and abdominal malignancy treatments.^1^, ^2^, ^3^ Attempts to contend with organ deformation in image‐guided and adaptive radiotherapy often involve the implementation of deformable image registration (DIR) algorithms.^4^, ^5^, ^6^, ^7^, ^8^ One approach for calculation of cumulative doses in moving and deforming targets for both inter‐ and intrafraction effects is via the ‘dose‐warping’ technique, using DIR to redistribute dose before summation.^9^, ^10^, ^11^ Our previous work has demonstrated experimentally (using a deformable, three‐dimensional, dose‐integrating tissue‐equivalent dosimeter, ‘DEFGEL’^(12)^) that this can be performed accurately^(13)^ when optimized appropriately. Combining this technique with 4D CT scanning has made possible 4D dose calculation, which facilitates incorporation of temporal information pertaining to tumor motion and deformation.

Previous studies have focused primarily on the case of pulmonary lesions.^14^, ^15^, ^16^, ^17^ However, like the lung, the liver is an organ that undergoes significant deformation due to respiration.^5^, ^18^ Stereotactic body radiotherapy (SBRT) for treatment of liver lesions, in principle, involves high dose conformity to the target with minimal dose to the normal tissue due to choice of tight margins.^19^, ^20^ Nonetheless, liver SBRT is subject to the uncertainties introduced by delivering inherently inhomogeneous dose distributions to deforming and moving organs.^(21)^ Consequently, associated discrepancies between planned and delivered doses could result in the potential risk of reduced target coverage and/or increased dose to organs at risk, including the surrounding healthy liver.

The objective of this study is to quantify the extent of dosimetric differences between conventional 3D (static) dose calculation and path‐integrated quasi‐4D cumulative dose calculation, effected via DIR‐based dose‐warping techniques, in the context of liver SBRT. This is particularly relevant in terms of accurate retrospective analysis of outcome dose correlation, with regard to both tumor control and normal tissue exposure. For comparison between 3D and 4D calculations, we consider whether organ dose metrics derived from contours defined on the average intensity projection (AIP), or on a reference phase, provide the better approximation of the 4D values. In addition, considering the fact that the 4D approach is inherently more time‐consuming, we also explore the number of phases required to accurately represent the results of the nominal full 10‐phase calculation approach based on the use of all phases available from our routine imaging. We demonstrate that care must be exercised when using plan quality evaluation metrics designed for 3D approaches to assess 4D calculated dose distributions.

## II. MATERIALS AND METHODS

Plans were evaluated for four consecutive patients, with five lesions, treated with SBRT for liver metastases. These datasets exhibited a significant range of GTV geometric traits in terms of tumor sizes and locations, as well as various degrees of tumor motion and deformation (summarized in Table 1).

**Table 1:** Patient characteristics (tumor volume and motion range) and $D_{95}$ from dose prescription with fractionation schemes for each patient. GTV = gross tumor volume; $r_{3D}=\text{magnitude of}\;3\text{D displacement vector}$; $D_{95}=\text{prescribed dose to at least}\;95\%\;\text{of volume}$.

			*Motion Range (mm)*			
*Lesion*	*GTV* $\left({\text{cm}}^{3}\right)$	$r_{3D}$	*SI*	*AP*	*LR*	$D_{95}$ *(Gy)*
A (Patient 1)	22.7	8.8	7.5	4.6	0.9	43.7 (7.28Gy×6)
B (Patient 1)	12.4	10.6	10.5	0.5	1.0	41.7 (6.95Gy×6)
C (Patient 2)	54.5	7.5	7.5	0.0	0.1	46.3 (7.72Gy×6)
D (Patient 3)	6.7	16.6	16.5	1.8	1.2	54.5 (10.9Gy×5)
E (Patient 4)	86.2	10.7	10.5	1.6	1.1	44.5 (7.42Gy×6)

### A. Patient data acquisition using 4D CT imaging

Patients were imaged using respiratory correlated X‐ray CT (Brilliance CT Big Bore; Philips Medical System, Cleveland, OH). Patients were advised to perform free regular breathing, and a respiration signal was acquired via a pressure sensor fixed to the abdominal region by an elastic belt. Respiratory‐correlated 4D CT datasets were comprised of a total of 10 phases (the highest bin resolution — used as standard practice), acquired at equally spaced time intervals (0%–90% of respiratory period) across the entire breathing cycle. All phases of the 4D CT datasets were imported into the Eclipse treatment planning system (Varian Medical Systems, Palo Alto, CA) for dose calculations.

### B. Conventional 3D treatment planning

Gross tumor volumes (GTVs) were delineated on the two extreme phases (end‐expiration and end‐inspiration). An internal target volume (ITV) was defined in accordance with an established target volume concept^22^, ^23^ as the intersection of GTV volumes at the extreme phases (end‐inspiration and end‐expiration) with no expansion for the CTV margin. A 5 mm margin was added for generation of the planning target volume (PTV). The liver and other normal anatomic structures were contoured at the end‐expiration phase, which was chosen as the reference phase where the smallest tumor motion and consequently minimum motion artifacts would be expected.^1^, ^8^ The ‘original’ (conventional 3D static) dose distribution was calculated using the average intensity projection (AIP). The normal liver is defined in the conventional manner (i.e., total liver minus ITV in the AIP, and total liver minus GTV in the reference phase). Treatment was planned for a Varian 21EX (Varian Medical Systems), equipped with a multileaf collimator (5 mm leaf width). Between seven and nine fields (coplanar/noncoplanar) were used for SBRT delivery via 6 MV photons at 600 MU/min. Dose was calculated using the Eclipse analytical anisotropic algorithm (AAA) with a grid size of 3 mm. Total doses of 42 Gy in 6 fractions or 50 Gy in 5 fractions were prescribed to the 80% covering isodose, typically achieving a minimum covering isodose between 80%–87% (i.e., maximum doses of 115%–125%). The resultant prescribed dose, delivered to at least 95% of the volume of interest (VOI) in the PTV, is shown in Table 1.

In clinical practice, our group performs conventional 3D planning using contours derived from the AIP. However, contouring on the reference phase is an alternative and equally acceptable workflow practice, as described by others.^(24)^ In this study, we assess the implications of contouring with either methodology in terms of 4D dosimetric accuracy.

### C. Deformable image registration and quasi‐4D path‐integrated dose accumulation

The end‐expiration phase was identified for each patient and defined as the reference (target) image for the treatment plan. The 9 remaining phases were used as the source (moving) images. The latter were morphed to approximate the target image using the optical flow method of deformable image registration (DIR). Selection of the algorithm employed for this study was justified based on our previous study,^25^, ^26^ as well as those of other groups^15^, ^17^ who have similarly used it in the context of lung treatments. We have used the DIRART implementations^(27)^ of these algorithms as these are freely available in the public domain and, thus, the present approach may be easily reproduced by interested readers. Furthermore, the calculated deformation vector fields (DVF) are accessible to the user for application to dose warping, which may not necessarily be the case for commercial software algorithms.

**Figure 1:**
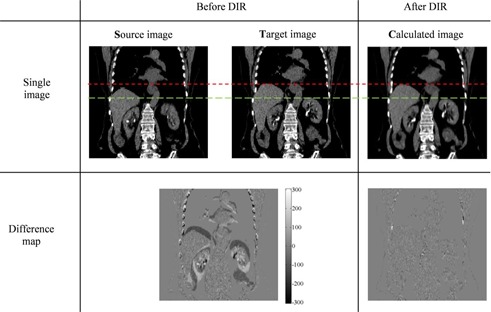
Example of deformable image registration (DIR) using the optical flow method shown in the coronal view (for Patient 1). The upper (red, short dash) and lower (green, long dash) lines show the alignment of the top of the liver and the kidneys respectively, before and after DIR to aid visual comparison of the images. A difference map before performing DIR illustrates the difference between the source and target images; the post‐DIR difference map compares the target and calculated images. The scale in the difference map is in HU.

Difference maps (between calculated and target images) were employed to evaluate the accuracy of the calculated deformations generated via DIR. The registration result for 2 extreme phases (end‐inspiration to end‐expiration) is shown in Fig. 1 as an example (coronal view). The high level of agreement (cross‐correlation of 0.9987) demonstrates the good performance of the optical flow method.

For each patient, the 3D static plan was applied individually to each of the 10 respiration phases of the 4D CT sets and recalculated. All planning parameters (e.g., prescribed monitor units, beam arrangement, leaf positions, isocenter position) remained unchanged. Deformation vector fields resulting from DIR were applied to the doses recalculated on each phase image using the TPS, to morph them back to the reference (end‐expiration) phase following anatomy changes. Difference maps between these warped doses and the reference dose calculated from 3D static plan were computed. The warped doses were equally weighted (as 10 phases of 4D CT image sets were acquired at equally spaced time intervals) to estimate the path‐integrated 4D cumulative dose distribution, which constitutes a more accurate approximation of the actual delivered dose than the 3D methodology.

### D. Evaluation of 3D static and 4D cumulative dose calculations

Dose‐volume histogram (DVH) analyses of the target volumes and the normal liver were performed. The GTV was defined from the end‐expiration phase. The conventional 3D static and quasi‐4D accumulated doses (referred to as $3D_{\text{REF}}$ dose and 4D dose, respectively) were compared using dose‐difference maps and in terms of dose homogeneity of target volumes. The dose to 98% of target volume ($D_{98}$, or “near‐minimum” dose) and the dose to 2% of the target volume ($D_{2}$ or “near‐maximum” dose) describe the range of dose that a target volume receives.^(28)^ The resultant ratio $D_{2}/D_{98}$ was adopted as a homogeneity index (HI),^(20)^ describing dose homogeneity that is related to the sharpness of the falloff in the DVH shoulder; an HI value of unity implies perfect homogeneity.

The biological effective dose (BED) was also calculated^(29)^ using the DVH‐based approach:

(1) $${\text{BED}}_{i}=nd_{i}\left[1+\frac{d_{i}}{\left(\alpha /\beta \right)}\right](\text{Gy})$$

where n=the number of fractions, $d_{i}=\text{the dose per fraction in bin i of the DVH}$, and the value α/β=2 Gy was used for late toxicity in the normal liver^(30)^ and the commonly used value α/β=10 Gy was applied for the tumor.^(31)^

Organ doses calculated using both the standard contouring on the reference phase ($3D_{\text{REF}}$) and on the AIP ($3D_{\text{AIP}}$) were compared to the 4D method ($4D_{\text{REF}}$, also contoured on the reference phase). Comparisons were undertaken in terms of the mean dose, $D_{\text{mean}}$, and near‐maximum dose, $D_{2}$. The former comparison ($3D_{\text{REF}}$ vs. $4D_{\text{REF}}$) is of course the focus of the work, and quantitatively assesses the dosimetric impact of motion and deformation on OAR doses. The latter comparison ($3D_{\text{AIP}}$ vs. $4D_{\text{REF}}$) allows identification of the preferred contouring methodology (i.e., whether contours defined on the AIP or reference phase provide the best approximation to the 4D case).

### E. Evaluation of 4D approach with < 10 phases

We also explored the number of phases required in the 4D evaluation to obtain an accurate estimate of the 10‐phase approach (with a view to minimizing calculation time). Accumulated dose distributions were investigated using fewer phases (p=2,3,5). For p=5, two alternative sets of 5 phases were used (labeled $5_{\text{even}}$ and $5_{\text{odd}}$). In each scenario, warped doses from the individual phases were averaged with equal weighting:

- $4D_{p=2}$ – two extreme phases (0% and 50%)
- $4D_{p=3}$ – 30%, 60%, and 90% phases
- $4D_{p=5,\text{even}}$ – 0%, 20%, 40%, 60%, and 80% phases
- $4D_{p=5,\text{odd}}$ – 10%, 30%, 50%, 70%, and 90% phases

Each of the above four‐dimensional cumulative dose calculations were compared to that calculated from the nominal full 10 phases ($4D_{p=10}$). Four‐dimensional evaluation using fewer than 10 phases was performed by comparing DVHs: the ratio of the fractional volumes was considered over the dose sub‐range $D_{98}–D_{50}$ for the target (PTV) and normal liver.

## III. RESULTS

Table 1 lists the patients' characteristics, including the tumor sizes and motion ranges, where the latter was defined as the displacement between the GTV centroids on the 2 extreme respiratory phases.

**Figure 2:**
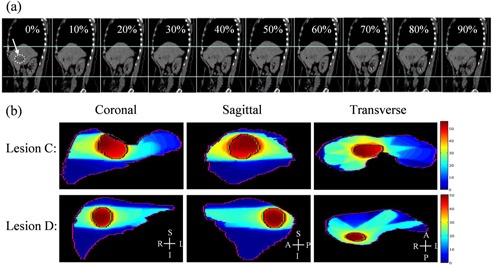
Example of sagittal planes (a) generated from the 4D CT sets for Lesion B. The arrow at 0% indicates the target region (indicated with a dotted line). The upper and lower horizontal lines of the figure are placed to guide the eye and help indicate the deformation of the liver and the right kidney. Dose distribution in liver (b): the planning target volumes (PTV) are indicated for Lesion C (the largest volume with the smallest motion) and Lesion D (the smallest volume with the largest motion) in three planes.

The tumor displacement (3D vector magnitude) due to breathing motion varied from 7.6 mm (Lesion C, with the largest volume) to 16.6 mm (Lesion D, with the smallest volume). As an example of an intermediate magnitude of tumor displacement, Fig. 2(a) shows sagittal views of Patient 1, generated from 10 phases of 4D CT sets through the same plane. Minor motion artifacts were observed, such as surface discontinuities, especially at the edge of the liver at midventilation phases; however, these did not influence the results as none of the target volumes incorporated regions exhibiting artifacts.

Figure 2(b) presents dose distributions in three orthogonal planes encompassing the maximum doses for Lesion C and Lesion D, illustrating the dose gradient in the PTV. The existence of the dose gradient, combined with the impact of deformation and motion, is evidence that discrepancies will exist between the 3D and 4D dose calculations. Consequently, this could imply overestimated dose conformity and tumor control for typical planning methodologies.

### A. Comparison of warped doses phase by phase

**Figure 3:**
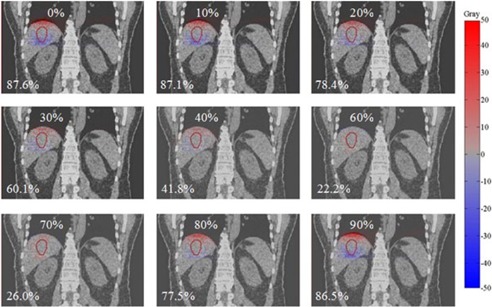
An example of dose‐difference maps between warped doses from each phase (0% to 90%) and the reference phase dose for Lesion D. The percentage in the bottom‐left corner of each figure indicates the maximum point dose difference inside the liver. Hot (red) and cold (blue) spots indicate positive and negative differences, respectively.

Difference maps between warped doses for each phase and the reference dose calculated from 3D static plan were computed. Dose‐difference maps of a coronal slice are shown in Fig. 3 for Lesion D, which exhibited the smallest tumor size with the largest displacement due to breathing motion amongst all five lesions. The value in the bottom‐left corner of each figure indicates the maximum point‐dose difference inside the liver. As expected, dose differences are greatest for the greatest motion. These maximum differences ranged from ~22% to ~88% with respect to the reference plan. The warped doses were equally weighted to estimate the path‐integrated 4D cumulative dose distributions (as shown in the next section).

### B. 4D evaluation of target volume doses: $3D_{\text{REF}}$ dose vs. 4D dose

**Figure 4:**
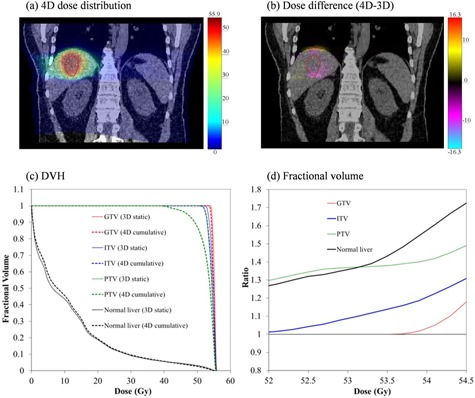
Illustration of 4D evaluation of 3D plan: (a) 4D calculated dose distribution for Lesion D (coronal plane shown); (b) dose difference between 3D and 4D in the same plane; all units of dose are gray; (c) dose‐volume histograms for targets and normal liver: solid and dashed lines illustrate 3D and 4D dose calculations, respectively; (d) the ratio of 3D‐calculated fractional volume relative to the 4D calculation over the $D_{98}$ to $D_{50}$ range derived from the ${\text{PTV}}_{3D}$ histogram (red = GTV, blue = ITV, green = PTV, and black = normal liver).

An example of the difference between $3D_{\text{REF}}$ and 4D dose computation is given in Fig. 4, which shows both a dose‐difference map and DVH differences. These results illustrate that the conventional 3D dose calculation overestimated dose for the targets (particularly PTV), whilst underestimating the dose to the normal liver, compared to the quasi‐4D dose calculation.

Table 2 summarizes the dose‐volume relationships for target volumes in the $3D_{\text{REF}}$ and 4D dose calculations, for all lesions studied. In the case of the 4D calculation, ${\text{BED}}_{\text{mean}}$ (4D) of the PTV was 1.5% to 8% lower than the 3D calculation. Furthermore, the conventional 3D approach overestimates the homogeneity. Specifically, the HI were on average over 1%, 4%, and 17% lower in the 3D case for the GTV, ITV, and PTV, respectively. The volumes ${\text{GTV}}_{+5\text{mm}}$ show a similar trend as ITV/PTV results, receiving less dose and poorer homogeneity than predicted by the 3D calculation (see Table 3). This indicates that the GTV dose may be well estimated using conventional 3D dose calculation, while the PTV dose may be significantly overestimated. For our patient cohort, the conventional method of 3D dose calculation overestimates dose homogeneity by up to ~24%, particularly in the high‐dose gradient region around the PTV margin. Healthy liver tissue adjacent to the PTV could move in and out of the treatment beam field over the breathing cycle, resulting in undesired dose to healthy liver and a reduction in dose conformity to the PTV. In quantifying such effects, the 4D methodology clearly demonstrates its advantage.

**Table 2:** Comparison between conventional 3D dose calculation and path‐integrated 4D cumulative dose calculation in target volumes; dose in Gy. HI is dimensionless and 3D refers to the dose for $3D_{\text{REF}}$.

*Lesion*	*Target (volume in* ${\text{cm}}^{3}$)	$D_{\text{mean}}$ *(3D / 4D)*	${\text{BED}}_{\text{mean}}$ *(3D / 4D)*	$D_{2}$ *(3D / 4D)*	$D_{98}$ *(3D / 4D)*	*HI (3D / 4D)*	${\text{HI}}_{3D}/{\text{HI}}_{4D}$ ($={\text{HI}}_{3D/4D}\%$)
A	GTV (22.7)	45.3 / 44.9	79.5 / 78.5	46.8 / 46.7	43.1 / 42.7	1.08 / 1.09	99.1
	ITV (31.1)	45.1 / 44.7	79.0 / 78.0	46.7 / 46.7	42.6 / 38.9	1.10 / 1.20	91.7
	PTV (66.4)	44.0 / 43.4	76.3 / 74.8	46.7 / 46.7	36.8 / 30.9	1.27 / 1.51	84.1
B	GTV (12.4)	42.7 / 40.4	73.1 / 67.6	43.4 / 43.2	41.1 / 39.8	1.06 / 1.08	98.2
	ITV (17.9)	42.1 / 39.0	71.6 / 64.4	43.3 / 43.2	36.9 / 33.8	1.17 / 1.28	91.4
	PTV (39.3)	41.1 / 37.8	69.2 / 61.7	43.3 / 43.2	31.7 / 26.4	1.37 / 1.64	83.5
C	GTV (54.5)	48.2 / 47.8	86.9 / 85.9	50.2 / 49.8	46.1 / 45.6	1.08 / 1.09	99.7
	ITV (87.0)	48.0 / 47.4	86.4 / 84.8	50.2 / 49.8	44.7 / 44.1	1.12 / 1.13	99.1
	PTV (168.7)	47.4 / 46.3	84.8 / 82.0	50.1 / 49.8	42.8 / 36.6	1.17 / 1.36	86.0
D	GTV (6.68)	55.3 / 55.0	116.5 / 115.5	55.8 / 55.6	54.3 / 53.9	1.02 / 1.03	99.6
	ITV (21.28)	54.8 / 54.4	114.9 / 113.6	55.7 / 55.5	53.0 / 52.2	1.05 / 1.06	98.8
	PTV (71.36)	52.7 / 50.7	108.2 / 102.1	55.6 / 55.4	46.3 / 35.3	1.20 / 1.57	76.5
E	GTV (86.16)	46.2 / 45.0	81.8 / 78.8	46.7 / 45.9	43.8 / 41.9	1.07 / 1.08	99.1
	ITV (100.5)	46.0 / 44.8	81.3 / 78.3	46.6 / 45.8	43.2 / 41.2	1.08 / 1.11	97.0
	PTV (187.24)	44.3 / 42.4	77.0 / 72.4	46.3 / 45.4	38.2 / 31.0	1.21 / 1.46	82.8

3D = three‐dimensional dose calculation; 4D = four‐dimensional dose calculation; $D_{\text{mean}}=\text{mean dose}$; ${\text{BED}}_{\text{mean}}=\text{biologically effective mean dose}$; $D_{2}=2\%\;\text{near}–\text{maximum dose}$; $D_{98}=98\%\;\text{near}–\text{minimum dose}$; HI = homogeneity index (the ratio of $D_{2}$ to $D_{98},D_{2}/D_{98}$); ${\text{HI}}_{3D/4D}\%$ = the percentage ratio of HI for 3D to 4D (${\text{HI}}_{3D}/{\text{HI}}_{4D}$). All doses in Gy.

**Table 3:** Comparison between conventional 3D dose calculation and path‐integrated 4D cumulative dose calculation in the volume of ${\text{GTV}}_{+5\text{mm}}$ margin.

${\text{GTV}}_{+5\text{mm}}$	*Vol*	$D_{\text{mean}}$		${\text{BED}}_{\text{mean}}$		$D_{2}$		$D_{98}$		*HI*		${\text{HI}}_{3D}/{\text{HI}}_{4D}$
*Lesion*	*(cc)*	*3D*	*4D*	*3D*	*4D*	*3D*	*4D*	*3D*	*4D*	*3D*	*4D*	*%*
A	46.4	44.4	42.8	77.3	73.3	46.6	46.4	40.4	37.6	1.15	1.23	93.5
B	29.2	42.0	39.8	71.4	66.2	43.3	43.2	38.3	34.0	1.13	1.24	90.9
C	100.4	47.8	47.5	85.9	85.0	49.6	49.8	45.3	44.8	1.09	1.11	98.5
D	18.6	54.8	54.5	114.9	113.9	55.6	55.5	53.1	52.3	1.05	1.06	98.7
E	143.3	45.3	43.6	79.5	75.3	46.6	45.9	40.6	35.9	1.15	1.28	89.8

3D = three‐dimensional dose calculation; 4D = four‐dimensional dose calculation; $D_{\text{mean}}=\text{mean dose}$; ${\text{BED}}_{\text{mean}}=\text{biologically effective mean dose}$; $D_{2}=2\%\;\text{near}–\text{maximum dose}$; $D_{98}=98\%\;\text{near}–\text{minimum dose}$; HI = homogeneity index (the ratio of $D_{2}$ to $D_{98},D_{2}/D_{98}$); ${\text{HI}}_{3D/4D}\%$ = the percentage ratio of HI for 3D to 4D (${\text{HI}}_{3D}/{\text{HI}}_{4D}$); ${\text{GTV}}_{+5\text{mm}}=\text{GTV}$ plus 5 mm margin. All doses in Gy.

Consistent with expectation, it is worth emphasizing that the smaller volume with larger motion (Lesion D) yielded a greater discrepancy between 3D and 4D dose calculations than the larger volume with smaller motion (Lesion C), in agreement with previous findings.^(14)^ Results in this study highlight that while ITV/PTV concepts ensure GTV coverage, the evaluation of dose coverage ($V_{x}$ and $D_{x}$) and homogeneity in these volumes are not necessarily appropriate indicators of plan quality for 4D evaluation of 3D plans.

### C. 4D evaluation of organs at risk: 3DREF & 3DAIP doses vs. 4D dose

**Figure 5:**
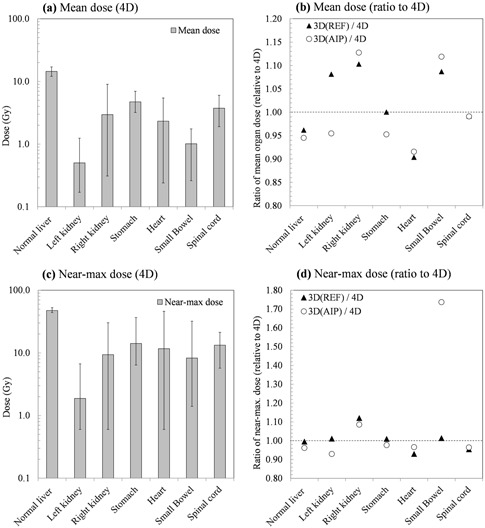
Comparison of 3D and 4D dose calculations to organs at risk. The data presented are the average over all patients studied. (a) The mean OAR doses; the error bars indicate maximum and minimum observed values. (b) The ratio of the mean dose as calculated via conventional 3D methods relative to the 4D case; shown for both contouring on the reference phase and on the AIP. (c) The near‐maximum dose ($D_{2\%}$); the error bars indicate maximum and minimum observed values. (d) The ratio of the near‐maximum dose as calculated via conventional 3D methods relative to the 4D case; shown for both contouring on the reference phase and on the AIP.

**Figure 6:**
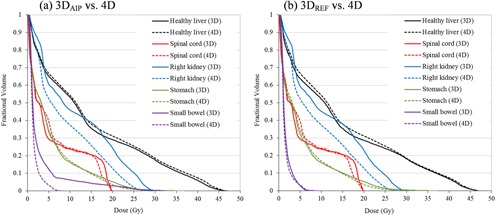
An example of dose‐volume histogram (DVHs) comparisons for critical organs in Patient 1: (a) $3D_{\text{AIP}}$ vs. 4D, (b) $3D_{\text{REF}}3$ vs. 4D. Solid and dashed lines illustrate the results calculated from 3D static and 4D cumulative dose calculation, respectively (black = normal liver, red = spinal cord, blue = right kidney, green = stomach, and purple = small bowel).

Figure 5 summarizes dose‐volume relationships for critical organs such as normal liver, spinal cord, right kidney, and duodenum in both dose calculation schemes. For normal liver, there is an observable trend such that the mean doses calculated from the 3D static dose calculation were up to 8% lower compared to 4D cumulative dose calculation. Doses to other organs are low and, consequently, differences between 3D and 4D methods are not as troubling in an absolute sense (see Fig. 6). This illustrates that reduced doses in target volumes due to organ motion/deformation lead to increased doses to the normal liver which encompass the entire tumor volume.

### D. Evaluation of 4D approach with < 10 phases

**Figure 7:**
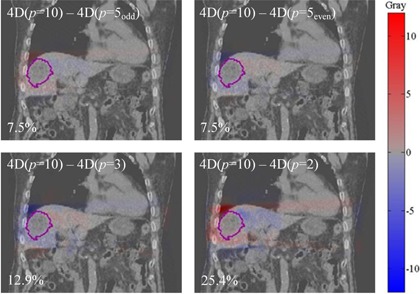
Dose‐difference maps between 4D doses calculated with different numbers, p, of phases ($p=2,3,5_{\text{even}}$ and $5_{\text{odd}}$) and the standard p=10 phases. The example is shown for Lesion E, in which the PTV is contoured. The maximum point‐dose difference is shown as a percentage in the bottom‐left of each panel. Hot (red) and cold (blue) spots indicate positive and negative differences, respectively.

Although 10 phases are generally required to adequately approximate the motion of a typical tumor due to respiration,^(32)^ using fewer phases would reduce calculation time and is consequently worth exploring in terms of accuracy. Figure 7 shows, for Lesion E, the coronal view of the dose‐difference maps between 4D cumulative dose calculated from the full 10 phases and that calculated from various subsets of phases ($p=2,3,5_{\text{even}}$, and $5_{\text{odd}}$). Doses in all cases were mapped back onto the reference image set (end‐expiration).

The maximum point dose difference in the PTV, displayed as a percentage in the bottom‐left corner of each figure, varied from 7.5% to 25.4%. As expected, this result illustrates that the more phases used for 4D dose calculation, the better the approximation of the delivered dose (p=10). These point‐wise discrepancies, however, do not generate a significant difference in DVH between the calculation schemes, as shown in Fig. 7.

**Figure 8:**
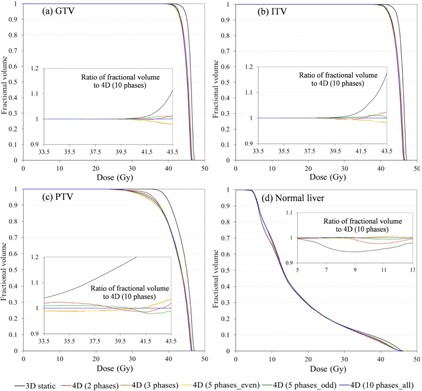
DVH for targets and normal liver in Lesion E: (a) GTV, (b) ITV, (c) PTV, and (d) normal liver. Various numbers of phases ($p=2,3,5_{\text{even}},5_{\text{odd}}$, and 10) were used for the 4D dose calculation. The 3D dose calculation is depicted as a black line and the 4D dose calculation as colored lines; p=2 phases (red); p=3 (orange); $p=5_{\text{even}}$ (orange); $p=5_{\text{odd}}$ (green); p=10 (blue). The inset figures show the ratio of fractional volume relative to the $4D_{p=10}$ calculation over the $D_{98}$ to $D_{50}$ interval derived from the $4D_{p=2}$ PTV and normal liver histograms.

Figure 8 shows DVH for targets and normal liver for Lesion E. The inset figure panels are ratios of 4D calculations of different numbers of phases ($p=2,3,5_{\text{even}},5_{\text{odd}}$) relative to the standard number of phases (p=10). The most striking observation one makes from this figure is that even the use of as few as 2 phases results in a more accurate estimate of the dose than the conventional 3D calculation. In this case (Lesion E), the $4D_{p=3}$ dose or even $4D_{p=2}$ give a good approximation of the full 10‐phase dose for the PTV, as well as GTV, ITV and normal liver; agreement within 3% is demonstrated over the $D_{98}$ to $D_{50}$ interval.

**Figure 9:**
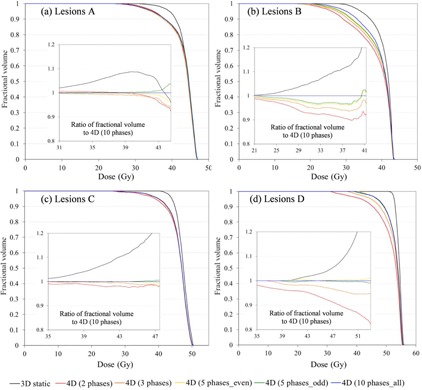
DVHs for PTV: (a) Lesion A; (b) Lesion B; (c) Lesion C; (d) Lesion D. Inserts show the ratio of fractional volume relative to the $4D_{p=10}$ calculation over the $D_{98}$ and $D_{50}$ interval derived from the $4D_{p=2}$ histogram for each lesion. All lines are as described for Figure 8.

For the other patients in this study, generally at least 3 phases are required to achieve a reasonable approximation of the delivered dose as represented by the 10‐phase set. This is shown in Fig. 9, which depicts DVH of the PTV for Lesions A–D, respectively; the insets are ratios to the 10 phase case. In these cases, 4D dose calculation using two extreme phases yielded differences of up to 10% and 17% for Lesion B and Lesion D, respectively (large motions with small target volumes), compared to the full 10‐phase method. In all cases, using 3 phases was enough to limit differences to less than 5%.

## IV. DISCUSSION

Clinical application of dose‐warping techniques is a contentious topic as reflected by, for instance, a recent point‐counterpoint article and correspondences published by *Medical Physics* which raised the question, ‘Is it appropriate to “deform” dose along with deformable image registration in adaptive radiotherapy?’^(33)^ Although the answer is not likely to be without complexity, a number of published studies have shown the applicability of dose‐warping techniques.^9^, ^10^, ^11^, ^14^, ^15^, ^16^, ^17^ We have previously corresponded to the point‐counterpoint article^34^, ^35^ following earlier work in which we demonstrated an experimental validation of the dose‐warping technique, as well as accurate performance of deformable registration algorithms.^(13)^ These studies have shown dose warping may be justified for small deformations in particular, and those that do not involve significant density changes.

The results we describe are consistent with other work undertaken concurrently with the present study,(24) which found that the GTV dose is estimated with sufficient accuracy using conventional 3D methods (contoured on the reference phase) when compared to the 4D approach. The results indicate that the mean dose to the PTV is consistently over estimated by the 3D approach (Table 2) by, on average, 4%. The near minimum dose (received by at least 98% of the volume) is more notably affected, while the near maximum dose in each volume is similar, regardless of whether 3D or 4D methods are used. Unexpected deficiencies in dose coverage at the periphery may result in the risk of inadequate tumor control. As one might expect, the greater consequences — in terms of absolute dose difference between 3D and 4D — occur for untargeted organs at risk (OAR). Even though absolute dose discrepancies are small, inaccurate estimation of healthy organ doses may have consequences, in terms of correlation with clinical outcomes and estimation of tissue complication risk.

It is not surprising that 4D evaluation of dose distributions identifies poorer coverage of target volumes such as the PTV — particularly where this incorporates an ITV enveloping the tumor motion excursion. One must be cautious in the apparent solace provided by GTV coverage metrics. The PTV concept is designed to compensate for other uncertainties beyond the motion identified in 4D imaging. Additionally, the 4D dose calculation is not a retrospective treatment evaluation, but an a priori plan evaluation. Clinically, the coverage of the PTV is still the planning objective, as it must be — underdosing the PTV risks undertreating the GTV in the presence of other uncertainties associated with patient setup, breathing profile changes, and anatomical changes since simulation. The 4D calculation approach provides improved understanding of the dose distributions being planned and delivered, but this new approach creates a demand for new, well‐understood plan quality metrics.

In 3D calculations, contours defined on the full expiration phase (REF) yield better dose estimates, in particular, for healthy liver than AIP contours. Although the two contouring methods (AIP and REF) for conventional 3D dose calculation result in significant differences in predicted healthy organ doses (several tens of percent), there was ultimately no strong trend for over‐ or underprediction of doses to particular organs. This negates the possibility of applying generic correction factors (or similar approaches) to doses estimated using AIP‐based 3D methods, and implies that, particularly for sensitive patient groups,^36^, ^37^, ^38^, ^39^ the 4D method ought to be employed. In the long term, it would be preferable for the 4D approach to be employed for all patients, such that accurate dose‐outcome correlation can be recorded and accurate tissue complication probabilities be established.

The conventional 3D dose calculation approach overestimates homogeneity by up to ~24%, occurring primarily in the high‐dose gradient region around the PTV margin. The GTV is of course less affected, though ${\text{HI}}_{3D/4D}$ of 98% nevertheless reflects an overestimate of homogeneity. Note that ${\text{HI}}_{3D/4D}$ of up to 10% for the volume of ${\text{GTV}}_{+5\text{mm}}$ margin highlights potential dosimetric misinterpretations from 3D conventional SBRT planning. There are of course arguments^40^, ^41^, ^42^, ^43^ for and against the ‘necessity’ of dose homogeneity, or at least the prioritization thereof but, regardless of the philosophy to which one subscribes, there is the unarguable necessity to know whether or not the dose is homogeneous.

Extending these results to other cases, one would expect even more pronounced effects in the case of very small fields, lung tumors, and high‐energy treatments.^44^, ^45^, ^46^ It is worth noting that ${\text{HI}}_{3D/4D}$ of the PTV was well correlated to the motion–volume relationship (see Table 1); a greater ratio of motion to volume (i.e., smaller volume with larger motion) yields greater ${\text{HI}}_{3D/4D}$ than a larger volume with smaller motion. In other words, where the volume is small and the motion is large, the 3D approach results in poor homogeneity estimation, whereas in the converse case it may not be necessary to pursue the 4D method, and the conventional 3D planning based on an AIP may be sufficient.

Since such 4D calculation is relatively resource intensive — increasing proportionally to the number of datasets analyzed — we investigated alternative reduced phase binned solutions. The objective of this was to determine whether fewer than the typical 10‐phase 4D CT dataset is sufficiently accurate. Together, the magnitude of the 3D motion vector and the GTV volume may be suitable indicators facilitating determination of whether a large number of phases is necessary for the 4D methodology and deserves further investigation. Notably, we found that for our patient cohort 4D dose calculation with as few as 3 phases was a reasonable approximation of the standard 10‐phase approach. Quantitatively, agreement was found to be within 5% over the $D_{98}$ to $D_{50}$ intervals in the PTV DVH for all lesions. The authors regard 5% deviation as a borderline for acceptability when accounting for other uncertainties specific to the treatment technique. Nevertheless, the implication of this finding is that a strategy of utilizing an abbreviated dose‐accumulation process warrants further investigation. It would be potentially useful, as resource restrictions are likely to serve as a barrier to clinical implementation of nonadaptive 4D dose calculation.

## V. CONCLUSIONS

In this work, we have investigated the potential limitation of conventional 3D dose calculation methods for liver SBRT by implementing nonadaptive 4D cumulative dose calculation and comparing it with the former. In this study, conventional 3D planning based on an AIP (i) overestimated target doses in ITV & PTV, (ii) underestimated healthy liver dose and poorly predicted doses to proximate OAR, and (iii) overestimated target dose homogeneity by up to 9% in ITV and 24% in PTV.

This has obvious implications for treatment plan evaluation, retrospective plan analysis, and outcome correlation. The quasi‐4D methodology described here provides improved dose distribution estimates and, therefore, additional information for clinical decision‐making. The familiar descriptors of ITV/PTV coverage and dose homogeneity may not be appropriate indicators of plan quality for 4D evaluation of 3D plans. Where full 4D dose calculations are not performed, contouring on the end‐expiration phase gave a better estimate of the 4D dose in the case of healthy liver, but otherwise contouring on the AIP was equally suitable. For the patients studied, using as few as 3 phases provided a good approximation of the full 10‐phase calculation for the quasi‐4D methodology, and warrants further investigation as a potential avenue for workload reduction.

## ACKNOWLEDGMENTS

This work is supported by an RMIT University Research & Innovation Emerging Researcher Industry Award (Dr. R. Franich).
